# An Adaptive Multivariate Two-Sample Test With Application to Microbiome Differential Abundance Analysis

**DOI:** 10.3389/fgene.2019.00350

**Published:** 2019-04-24

**Authors:** Kalins Banerjee, Ni Zhao, Arun Srinivasan, Lingzhou Xue, Steven D. Hicks, Frank A. Middleton, Rongling Wu, Xiang Zhan

**Affiliations:** ^1^Department of Public Health Sciences, Pennsylvania State University, Hershey, PA, United States; ^2^Department of Biostatistics, Johns Hopkins University, Baltimore, MD, United States; ^3^Department of Statistics, Pennsylvania State University, University Park, PA, United States; ^4^Department of Pediatrics, Pennsylvania State University, Hershey, PA, United States; ^5^Department of Neuroscience, State University of New York Upstate Medical University, Syracuse, NY, United States

**Keywords:** adaptive microbiome differential analysis (AMDA), maximum mean discrepancy (MMD), multivariate two-sample test, permutation, subset testing, taxa-set

## Abstract

Differential abundance analysis is a crucial task in many microbiome studies, where the central goal is to identify microbiome taxa associated with certain biological or clinical conditions. There are two different modes of microbiome differential abundance analysis: the individual-based univariate differential abundance analysis and the group-based multivariate differential abundance analysis. The univariate analysis identifies differentially abundant microbiome taxa subject to multiple correction under certain statistical error measurements such as false discovery rate, which is typically complicated by the high-dimensionality of taxa and complex correlation structure among taxa. The multivariate analysis evaluates the overall shift in the abundance of microbiome composition between two conditions, which provides useful preliminary differential information for the necessity of follow-up validation studies. In this paper, we present a novel **A**daptive multivariate two-sample test for **M**icrobiome **D**ifferential **A**nalysis (**AMDA**) to examine whether the composition of a taxa-set are different between two conditions. Our simulation studies and real data applications demonstrated that the AMDA test was often more powerful than several competing methods while preserving the correct type I error rate. A free implementation of our AMDA method in R software is available at https://github.com/xyz5074/AMDA.

## 1. Introduction

The human microbiome, referred as the aggregate of microorganisms that resides on or within any human tissues and biofluids, has recently gained substantial scientific interest due to its vital role in many human health and disease conditions, including but are not limited to obesity (Turnbaugh et al., 2009), type 2 diabetes (Qin et al., 2012), rheumatoid arthritis (Zhang et al., 2015), inflammatory bowel disease (Morgan et al., 2015), bacterial vaginosis (Mitchell et al., 2017), and colorectal cancer (Louis et al., 2014). High-throughput sequencing technologies have revolutionized microbiome research by allowing culture-free profiling of entire microbiome community. For the most part, 16S rRNA gene amplicon sequencing and metagenomics shotgun sequencing are routinely used for quantitative characterization of microbiome composition (Wang and Jia, 2016). Although data produced by high-throughput sequencing has been proven extremely useful for quantification of microbiome composition, yet appropriate analysis of such microbiome composition data is still computationally and statistically challenging due to some technical aspects of the data, including high-dimensionality, count or compositional data structure, sparsity (zero-inflation), over-dispersion, among others.

In many microbiome studies, the investigators are often interested in studying how the abundance of microbiome is related with clinical characteristics of the samples, such as health/disease status, smoking status, or dietary habit (high-calorie or low-calorie). That is, many studies attempt to detect differentially abundant microbiome features (species/OTUs) between two predefined classes of samples, where a microbiome feature is considered differentially abundant, if its mean proportion is significantly different between two conditions. This type of analysis can improve understanding the pathology of the disease from a microbiome perspective and potentially lead to preventive or therapeutic strategies (Virgin and Todd, 2011). Microbiome differential abundance analysis (MDA) is a direct analogy to differential expression analysis for gene expression and RNA-seq data, however, the distinct nature of microbiome data renders classic differential expression analysis methods such as DESeq (Anders and Huber, 2010) and edgeR (Robinson et al., 2010) inappropriate for microbiome data (McMurdie and Holmes, 2014; Weiss et al., 2017). Thus, new statistical methods for microbiome differential abundance analysis are desired.

Similar to individual gene-based and pathway-based differential expression analysis, there are two types of microbiome differential analyses: individual taxon-based univariate analysis and taxa set-based multivariate analysis. Along with the recent huge scientific interest in microbiome studies, many statistical methods for microbiome differential analysis have also been proposed (Sohn et al., 2015; Zhao et al., 2015; Zhang et al., 2016; Chen et al., 2017), with most of them focus on examining whether a single taxon is differentially abundant between two different conditions, followed by multiple testing correction methods adjusting for individual taxon *p*-values (e.g., the Benjamini-Hochberg/BH procedure, Benjamini and Hochberg, 1995). The control of False Discovery Rate (FDR) is necessary, as an excess of false discoveries may lead to costly follow-up validation studies on false positive taxa, which essentially are not differentially abundant. Despite their potential usefulness in identifying differentially abundant taxa, these individual analyses may suffer from the following inherent limitations. First, the type I error of an individual microbiome differential analysis may not be correct (Hawinkel et al., 2017). The BH procedure or its variant can control FDR when individual tests are either independent or under positive dependence assumptions (Benjamini and Hochberg, 1995; Benjamini and Yekutieli, 2001), while negative correlation among taxa abundance is common in microbiome data, especially for compositional data. It is possible that these BH procedures (Benjamini and Hochberg, 1995; Benjamini and Yekutieli, 2001) may fail to control FDR in presence of negative correlations (Hawinkel et al., 2017). Second, the high-dimensionality nature of microbiome data increases multiple correction burden of individual analyses, which reduces the power of detecting differentially abundant taxa. Third, as widely observed in literature, the performance of most individual microbiome differential analysis methods heavily rely on the normalization and/or transformation, leading to challenges in independent replication studies (McMurdie and Holmes, 2014; Sohn et al., 2015; Weiss et al., 2017).

An alternative approach to taxon-level microbiome differential analysis is to compare the microbiome composition at the level of taxa-set. Examples of such a taxa set can be either a group of OTUs belonging to the same upper-level taxonomic rank (e.g., phylum, class, order, family, or genus) or even all OTUs in the microbiome community. The multivariate-type microbiome differential analysis usually gains power by reducing the multiple testing correction burden and aggregating modest effects across multiple taxa. Moreover, the multivariate analysis is typically less sensitive to normalization/transformation compared to individual analysis as it has a much larger analysis unit. Motivated by this, many statistical methods for microbiome community-level analysis have been recently proposed (McArdle and Anderson, 2001; Zhao et al., 2015; Tang et al., 2016, 2017; Plantinga et al., 2017; Zhan et al., 2017a).

Despite of the potential power gain, a major critique of these existing multivariate microbiome analyses (e.g., differential analysis) is that the result of the test is global and is unable to identify specific taxon in the taxa-set that are differentially abundant. Besides the limitation in results' interpretation, it may also jeopardize the power of the test when the taxa-set contains many taxa that are not differentially abundant (Cao et al., 2017). To enhance both interpretation and power of existing multivariate analysis in the framework of MDA, we propose a two-stage Adaptive Microbiome Differential Analysis (AMDA) procedure, which first selects some putative taxa that are more likely to be differentially abundant between two conditions, and then examines the differential abundances of the selected taxa-set with a multivariate two-sample test using Maximum Mean Discrepancy (MMD) (Gretton et al., 2007, 2012). Since the test is applied to a subset of taxa that are more likely to be differentially abundant, permutations are used to establish statistical significance to avoid inflated type I error. Despite being a set-based multivariate test that does not target at identifying individual differentially abundant microbial taxa, the intermediate testing subset selection procedure in AMDA can provide useful information regarding the importance of individual taxon in the taxa-set. Simulation studies and real data applications demonstrate the potential usefulness of the new proposed AMDA method and show its superior performance over existing methods across a wide range of scenarios.

## 2. Materials and Methods

### 2.1. Data and Normalization

Assume that we have measured the microbiome abundances of a community of *p* taxa from *n*(= *n*_1_+*n*_2_) samples collected from two groups with sizes of *n*_1_ and *n*_2_, respectively. Here, the term community refers as a taxa-set, which typically consists of taxa from the same taxonomic rank such as genus, family, phylum, or bacteria kingdom. Let ${\text{X}}^{\left(k\right)}={\left(X_{1}^{\left(k\right)},\ldots ,X_{n_{k}}^{\left(k\right)}\right)}^{T}$ be the observed *n*_*k*_ × *p* OTU matrix for group *k*(*k* = 1, 2), where $X_{i}^{\left(k\right)}\left(i=1,\ldots ,n_{k};k=1,2\right)$ represents a *p* × 1 microbiome composition vector (subject to appropriate normalization or transformation). Suppose that, $X_{1}^{\left(k\right)},\ldots ,X_{n_{k}}^{\left(k\right)}\left(k=1,2\right)$ are two independent samples, from *p*-dimensional multivariate distribution with mean parameters **μ**^(1)^ and **μ**^(2)^, respectively. In many practical problems, the hypothesis of interest is to examine whether microbiome abundances are different under two different conditions, that is,

(1) $$H_{0}:\mu^{\left(1\right)}=\mu^{\left(2\right)}\;vs.\;H_{1}:\mu^{\left(1\right)}\neq \mu^{\left(2\right)}.$$

For microbiome data, due to the varying amount of DNA yielding materials across different samples, the count of microbiome sequencing reads can vary greatly from sample to sample. The normalization of the raw sequencing read counts to relative abundances makes the microbial abundances comparable across samples. Therefore, it is a common practice to analyze high-dimensional microbiome compositional data with a unit sum (Li, 2015). As such, applying standard statistical methods developed for unconstrained data to analyze microbiome composition data is usually underpowered and sometimes can render inappropriate results (Cao et al., 2017; Weiss et al., 2017).

A popular approach to relax the compositional constraint of microbiome data is to perform the statistical analysis through log-ratio transformations (Aitchison, 1982). In particular, the centered log-ratio transformation has been widely used among various form of log-ratio transformations (Cao et al., 2017; Zhao et al., 2018). Specifically, the centered log-ratio transformation $Z_{ij}^{\left(k\right)}$ of microbiome relative abundance $X_{ij}^{\left(k\right)}$ is defined as

(2) $$\begin{array}{l} Z_{ij}^{\left(k\right)}=\log\left(\frac{X_{ij}^{\left(k\right)}}{{\left(\Pi_{j=1}^{p}X_{ij}^{\left(k\right)}\right)}^{1/p}}\right),\;i=1,\ldots ,n_{k},j=1,\ldots ,p,\\ \;k=1,2. \end{array}$$

To avoid a zero relative abundance in Equation (2), as a common practice, a zero count is usually replaced by a pseudo count of 0.5 before the relative abundance normalization and centered log-ratio transformation (Li, 2015; Cao et al., 2017). For community-based multivariate differential abundance analysis, it has been shown that testing equality of two compositional vectors is equivalent to testing $H_{0}^{\prime }:\mu_{Z}^{\left(1\right)}=\mu_{Z}^{\left(2\right)}$ (Cao et al., 2017), where $\mu_{Z}^{\left(k\right)}$ is the mean of centered log-ratio transformed compositional vector $Z_{i}^{\left(k\right)}$, *i* = 1, …, *n*_*k*_ and *k* = 1, 2. We will develop our AMDA method based on these centered log-ratio transformed relative abundances in the rest of this paper.

### 2.2. A Multivariate Two-Sample Test Using Maximum Mean Discrepancy

Two-sample testing on the equality of two high-dimensional means has been well studied in the statistical literature (Bai and Saranadasa, 1996; Chen et al., 2010; Cai et al., 2014). These methods are typically not applicable to MDA analysis due to the following two reasons. First, existing methods usually assume normal data, which is not the case for microbiome compositional data. It has been observed that classic statistical methods developed for multivariate Gaussian data may fail for microbiome compositional data (Li, 2015; Cao et al., 2017; Zhao et al., 2018). Second, most existing methods require estimating the covariance matrix. Given the small or modest sample size in a typical microbiome study, the relatively large estimation error of covariance matrix probably deteriorates the performance of two-sample test, as observed in microbiome association tests (Zhan et al., 2017b, 2018).

An alternative approach to test hypothesis (Equation 1)is to use a non-parametric test that does not need to estimate the covariance matrix. One such test is the kernel-based maximum mean discrepancy (MMD) test (Gretton et al., 2007, 2012), originally proposed to examine whether the underlying distribution of two samples are identical. An MMD test first maps the two distributions into a reproducing kernel Hilbert space (RKHS) and then the maximum mean discrepancy metric between the two distributions is defined as the distance of their corresponding images in the RKHS. A good property about MMD is that, MMD is zero if and only if two distributions are identical when the RKHS is sufficiently rich (contain a large enough class of functions). Since the test can be used to examine equality of two multivariate distributions, it suffices for testing (Equation 1), that is, to examine the equality of the mean parameters of two underlying distributions.

In particular, the MMD statistic between two independent samples $X_{1}^{\left(1\right)},\ldots ,X_{n_{1}}^{\left(1\right)}$ and $X_{1}^{\left(2\right)},\ldots ,X_{n_{2}}^{\left(2\right)}$ is defined as

(3) $$\begin{array}{l} {\text{MMD}}^{2}=\frac{1}{n_{1}^{2}}\sum_{i=1}^{n_{1}}\sum_{j=1}^{n_{1}}k(X_{i}^{\left(1\right)},X_{j}^{\left(1\right)})+\frac{1}{n_{2}^{2}}\sum_{i=1}^{n_{2}}\sum_{j=1}^{n_{2}}k(X_{i}^{\left(2\right)},X_{j}^{\left(2\right)})\\ \;-\frac{2}{n_{1}n_{2}}\sum_{i=1}^{n_{1}}\sum_{j=1}^{n_{2}}k(X_{i}^{\left(1\right)},X_{j}^{\left(2\right)}), \end{array}$$

where *k*(·, ·) is a characteristic kernel (Gretton et al., 2007, 2012), which spans a RKHS which is sufficiently large that MMD is zero if and only if two samples are from the same underlying distribution. Examples of characteristic kernel include the Gaussian kernel and the Laplace kernel. Under the null hypothesis of identical distribution, the population-level MMD^2^ statistic is zero, and thus, a larger MMD^2^ statistic indicates a larger discrepancy between the two distributions. Asymptotically, MMD^2^ follows a mixture of $\chi_{1}^{2}$ distribution (Gretton et al., 2007, 2012). As observed in literature, the asymptotic mixture of $\chi_{1}^{2}$ distribution is typically not accurate for a statistic calculated from a small sample size, as frequently encountered in microbiome studies (Chen et al., 2016; Zhan et al., 2017b, 2018). A more accurate approach to establish significance is using resamplings (e.g., permuting the group label of each observation) (Wu et al., 2016).

### 2.3. An Adaptive Two-Sample Test for Microbiome Differential Abundance Analysis

A limitation of the aforementioned MMD test is that it equally utilizes information in all dimensions. When the signal is sparse, the MMD test typically has a low power due to the high degrees of freedom paid for many noise variables. The same phenomenon has been widely observed in the field of set-based genetic association studies (Cai et al., 2012; Pan et al., 2014, 2015; Zhan et al., 2015) and community-based microbiome association studies (Wu et al., 2016; Koh et al., 2017). There are in general two types of two-sample test of high-dimensional means. One is based on the sum of squares of mean differences of each dimension [e.g., MiRKAT proposed in Zhao et al., 2015], and the other is based on the largest componentwise mean difference (e.g., the max-type test proposed in Cao et al. (2017)). For microbiome differential abundance analysis, the max-type test tends to be more powerful when only a few taxa are truly differentially abundant. On the other hand, the MiRKAT-type test can be more powerful than the max-type test under the scenario of dense signals. In practice, the true underlying biological scenario is never known and thus adaptive methods for microbiome differential abundance analysis are desired.

A common adaptive approach in a multivariate association test or two-sample test is to assign different weights to variables so that important variables are up-weighted and non-informative variables are down-weighted (Cai et al., 2012; Pan et al., 2014, 2015; Wu et al., 2016; Koh et al., 2017). Yet it is often difficult to determine the optimal weights. Some authors propose another loop of permutations to combine multiple sets of weights, which may be computationally challenging since most adaptive tests already need permutations to establish significance (Pan et al., 2014, 2015). In this paper, we propose a different adaptive method, which tests the hypothesis in a selected subset of microbiome features. In other words, instead of applying the MMD test to all *p* taxa **X** = (*X*_1_, …, *X*_*p*_), we apply the test on a putative testing subset **X**_*S*_, where *S*⊂{1, …, *p*}. Our method can also be viewed as a weighted approach in the sense that a zero weight is assigned to a feature that is not selected in the testing subset, and an equal weight is assigned to each feature in the testing subset. We defer details of selecting such a testing subset to the next section and present our adaptive microbiome differential analysis (AMDA) procedure in Algorithm 1:

**Algorithm 1:** An adaptive two-sample test for microbiome differential abundance analysis

**Input:** A *n*×*p* microbiome composition matrix $\text{X}={\left(X_{1}^{\left(1\right)},\ldots ,X_{n_{1}}^{\left(1\right)},X_{1}^{\left(2\right)},\ldots ,X_{n_{2}}^{\left(2\right)}\right)}^{T}$ and a *n* × 1 group label vector *y* = (1, …, 1, 2, …, 2) associated with the microbiome compositions.
**Output:** A *p*-value for $H_{0}:\mu^{\left(1\right)}=\mu^{\left(2\right)}\text{ vs.}H_{1}:\mu^{\left(1\right)}\neq \mu^{\left(2\right)}$.
**Procedure:** Apply the centered log-ratio transformation Equation (2) to the microbiome composition matrix. Without loss of generality, we still use **X** to denote the centered log-ratio transformed data. Use the testing subset selection procedure described in section 2.4 to select a testing subset ${\text{X}}_{S}$ from **X**, and then calculate the MMD statistic using ${\text{X}}_{S}$ and *y*. Denote this statistic as $MMD_{obs}^{2}$. For *b* = 1, …, *B*, permute the group label of observations to obtain ỹ and use ỹ to repeat Step 2 with **X** and ỹ. Calculate the corresponding statistics as $MMD_{b}^{2}$ for *b* = 1, …, *B*. Calculate the final *p*-value as $pv=\frac{1}{B}\overset{B}{\underset{b=1}{\sum }}I\left[MMD_{b}^{2}\geq MMD_{obs}^{2}\right]$, where *I*[·] is the indicator function.

### 2.4. A New Permutation-Based Testing Subset Selection Procedure

There is a vast statistical literature on high-dimensional variable selection. Some famous examples include the lasso (Tibshirani, 1996) and the knockoff filter (Barber and Candès, 2015; Candes et al., 2018). The lasso has proven to be a versatile tool with nice asymptotic estimation and prediction properties, yet its performance under small sample size is not guaranteed. On the other hand, knockoff is able to select variables under FDR control with finite samples. But it tends to select a smaller set of variables with less false positives to achieve FDR control (see Table S1 in the online supplemental material). As a consequence, many signals are not selected by knockoff, typically leading to a less powerful test. Recall that, our ultimate goal is to construct a differential test with relatively high power. For this reason, we prefer a procedure that can select a testing subset that contains as many signals as possible. To achieve this goal, we propose the following permutation-based testing subset selection procedure.

We first randomly permute the row indices of matrix **X** (defined in Algorithm 1) and obtain a permuted microbiome composition matrix $\tilde{\text{X}}$. By the nature of its construction, $\tilde{\text{X}}$ is not related to outcome *y*. Next, a one-dimensional two-sample test (e.g., the Kolmogorov-Smirnov test) is applied to each dimension of **X** and $\tilde{\text{X}}$, and we denote the corresponding *p*-values as *p*_1_, …, *p*_*p*_ and ${\tilde{p}}_{1},\ldots ,{\tilde{p}}_{p}$, respectively. Because the dimension *p* is typically much larger than sample size in microbiome studies, we calculate the marginal *p*-values rather than joint *p*-values for testing subset selection. For a truly differentially expressed variable *X*_*j*_, as ${\tilde{X}}_{j}$ is not constructed to be outcome-related, it is expected that $p_{j}<{\tilde{p}}_{j}$. Hence, we select the testing subset as $S=\left\{j:p_{j}<{\tilde{p}}_{j}\right\}$ and conduct our MMD test based on the sub-design matrix ${\text{X}}_{S}$. Finally, as we are testing $H_{0}:\mu^{\left(1\right)}=\mu^{\left(2\right)}$ using microbiome features that are more likely to be differentially expressed, to avoid inflated type I error, resampling methods are required to establish the significance (see details in Algorithm 1).

It should be noted that the aforementioned permutation-based procedure is one way to achieve testing subset selection but not the only way, and it is possible to select testing subset ${\text{X}}_{S}$ using other methods such as lasso and knockoff. We conduct comprehensive simulation studies to compare the power of adaptive two-sample test using different testing subset selection procedures and report the results in the online Supplementary Material. As can be observed there, adaptive test based on our permutation-based procedure is more powerful than both lasso-based and knockoff-based tests, as both lasso and knockoff tend to miss more true signals for the sake of achieving sparsity (lasso) or FDR control (knockoff).

## 3. Results

### 3.1. Simulation Settings

A comprehensive simulation study has been conducted to compare the performance of AMDA to a wide range of existing microbiome association tests in the framework of microbiome differential abundance analysis. The five other tests evaluated in this simulation include the MiRKAT (Zhao et al., 2015), the original MMD test without testing subset selection (Gretton et al., 2007, 2012), the Quasi-Conditional Association Test/QCAT (Tang et al., 2017), the maximum-type (MAX) test based on the largest sample mean difference (Cao et al., 2017) and the optimal microbiome-based association test/OMiAT (Koh et al., 2017). AMDA, MiRKAT, MMD, QCAT, and MAX are a single test, while OMiAT takes advantage of two series of tests. One is the MiSPU tests (Wu et al., 2016) with different weighting schemes on each individual taxon in the taxa-set. The other is the MiRKAT tests with different kernel functions. The spirit of OMiAT can be easily implemented in AMDA, MiRKAT, and MMD by evaluating multiple kernels and taking the optimal kernel test with minimum *p*-value. We do not incorporate this strategy, for ease of presenting, and only evaluate the Gaussian kernel-based test for AMDA, MiRKAT, and MMD in this simulation. Correspondingly, we evaluate the OMiAT as the optimal of a series of MiSPU tests (without MiRKAT tests of different kernels) for fair comparison. With a slight abuse of notation, we still term this test as OMiAT, though it does not contain the MiRKAT component compared to the original one (Koh et al., 2017). Moreover, QCAT and MAX tests with asymptotic *p*-values are found to have inflated type I errors (data not shown). For this reason, we use permutations to calculate the MAX test *p*-value and the resampling option in the QCAT software (Tang et al., 2017) to calculate QCAT *p*-value. Finally, the permutation-based procedure is used to select testing subset in the intermediate stage of AMDA in this simulation. The performance of AMDA test based on other subset selection methods such as lasso and knockoff were evaluated in additional simulation studies presented in the online Supplementary Material.

We closely followed the simulation design of the MAX test (Cao et al., 2017) to generate microbiome relative abundances data using the logistic normal distribution (Atchison and Shen, 1980). We first simulated ${\text{W}}_{i}^{\left(k\right)}\sim N_{p}\left(\mu^{\left(k\right)},\Sigma \right)$ for *i* = 1, 2, …, *n*, *k* = 1, 2 and then calculated the microbiome relative abundances as $X_{ij}^{\left(k\right)}=\exp\left[W_{ij}^{\left(k\right)}\right]/\overset{p}{\underset{j=1}{\sum }}\exp\left[W_{ij}^{\left(k\right)}\right]$ and its centered log-ratio transformation $Z_{ij}^{\left(k\right)}$ according to Equation (2). Following the simulation design of MAX (Cao et al., 2017), the components of **μ**^(1)^ were drawn from a uniform distribution Unif(0,10) and we considered the banded covariance structure **Σ** = **D**^1/2^**AD**^1/2^, where **D** is a diagonal matrix with entries randomly drawn from Unif(1,3) and **A** has nonzero entries *a*_*jj*_ = 1, *a*_*j,j*−1_ = *a*_*j*−1,*j*_ = −0.5. Under the null model, we set **μ**^(2)^ = **μ**^(1)^. Under the alternative model, we randomly picked a subset S⊂{1,2,…,p} such that $\mu_{j}^{\left(2\right)}=\mu_{j}^{\left(1\right)}+e_{j}$, where *e*_*j*_ ~ *Unif*(−0.5, 0.5) for all j∈S. For the size of signal set S (number of taxa that are truly differentially abundant), we considered low, medium and high signal density levels: $p^{*}=|S|=10\%p$, 30*%p* and 50*%p* with the indices randomly chosen from {1, 2, …, *p*}. Throughout this simulation, we varied *n* = 50, 100, 200 with *n*_1_ = *n*_2_ = *n*/2 to investigate the test's performance under different sample sizes, and considered *p* = 50, 100, 200, 500 representing taxa-sets under different taxonomic ranks.

After the data were simulated, we applied AMDA, MAX, OMiAT, MMD, MiRKAT, and QCAT to examine the two-sample differences. The first three tests AMDA, MAX, OMiAT are adaptive in the sense that they either use a testing subset of the taxa (AMDA and MAX) or assign a different weight for each taxon in the set (OMiAT) to conduct the multivariate two-sample test. The Gaussian kernel (*k*(*x, y*) = exp{−||*x*−*y*||^2^/ρ}, where *x* and *y* are two microbiome compositional vectors) was used in AMDA, MMD, and MiRKAT with the shape parameter ρ selected as the median of sample pairwise Euclidean distance ||*x* − *y*||^2^. The type I error was evaluated using 5,000 replicates generated under the null model and the power of test was assessed with 1,000 replicates under the alternative model. Without loss of generality, we set the nominal significance level α = 0.05 throughout this simulation.

### 3.2. Simulation Results

**Figure 1:**
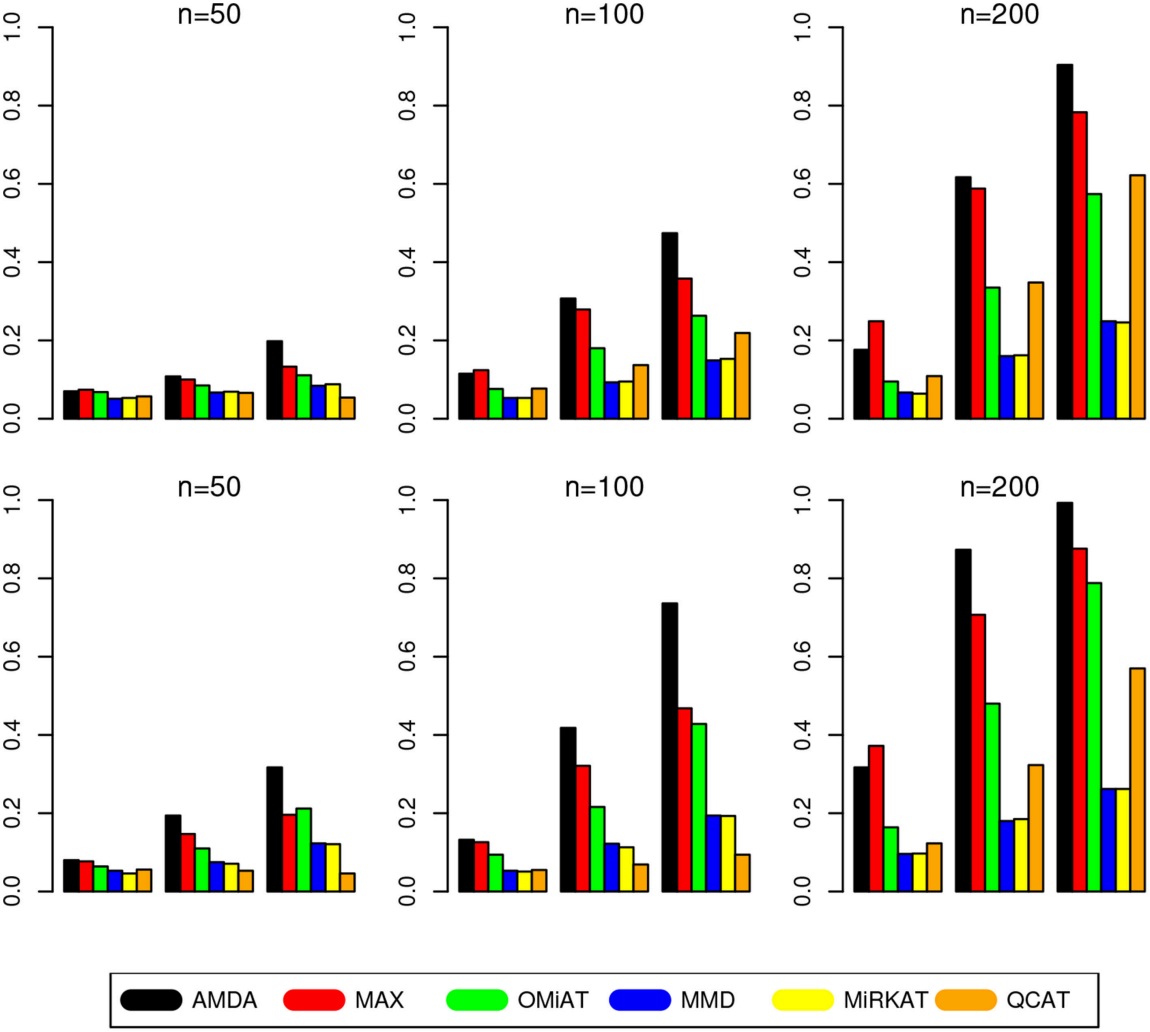
Empirical power of different tests under *p* = 50 (first row) and *p* = 100 (second row). The Y-axis represents the power and the X-axis represents the sparsity level at 10, 30, and 50%.

**Figure 2:**
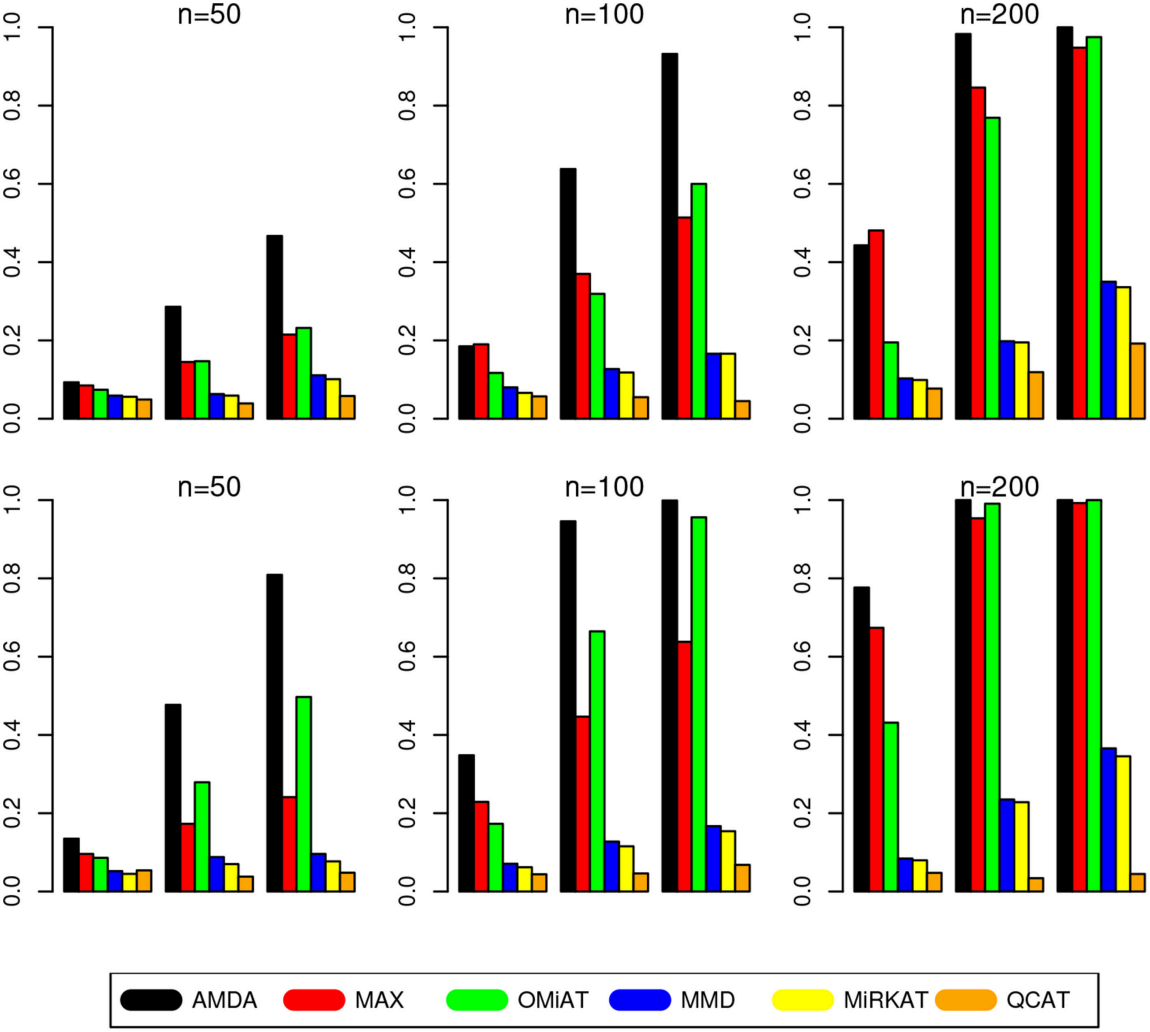
Empirical power of different tests under *p* = 200 (first row) and *p* = 500 (second row). The Y-axis represents the power and the X-axis represents the sparsity level at 10, 30, and 50%.

The type I error of different tests are reported in Table 1, where one can see that all tests have the correct type I error across all (*n, p*)-configurations. The power of different tests are reported Figure 1 (*p* = 50 and 100) and Figure 2 (*p* = 200 and 500). Since the effect size was arbitrarily chosen to avoid power saturation, we care about the relative power among different methods rather than their absolute magnitudes. As can be seen from both figures, adaptive tests (AMDA, MAX, and OMiAT) are consistently more powerful than the non-adaptive ones (MMD, MiRKAT, and QCAT). This is because the scenarios considered in our simulation studies are relatively sparse (p^*^/*p* ≤ 50%), and the adaptive tests can largely boost the power by treating variables (signals and noises) differently.

**Table 1:** Empirical type I errors of different tests for microbiome differential abundance analysis under nominal significance level α = 0.05.

**p**	**n**	**AMDA**	**MAX**	**OMiAT**	**MMD**	**MiRKAT**	**QCAT**
	50	0.0478	0.0478	0.0506	0.0516	0.0508	0.0436
50	100	0.0464	0.0458	0.0492	0.0536	0.0540	0.0488
	200	0.0504	0.0542	0.0530	0.0534	0.0548	0.0480
	50	0.0486	0.0478	0.0490	0.0434	0.0424	0.0532
100	100	0.0464	0.0494	0.0492	0.0544	0.0542	0.0478
	200	0.0524	0.0558	0.0514	0.0440	0.0424	0.0470
	50	0.0454	0.0498	0.0492	0.0438	0.0400	0.0490
200	100	0.0514	0.0476	0.0464	0.0530	0.0516	0.0538
	200	0.0464	0.0510	0.0506	0.0542	0.0530	0.0476
	50	0.0480	0.0464	0.0504	0.0556	0.0442	0.0474
500	100	0.0540	0.0544	0.0566	0.0570	0.0498	0.0468
	200	0.0556	0.0576	0.0456	0.0490	0.0442	0.0336

*Results are averaged over 5,000 replicates*.

Among three non-adaptive tests, MMD and MiRKAT have similar power under each scenario. On the other hand, QCAT has the highest power when the dimension of taxa-set is relatively low (Figure 1) especially when the sample size is relatively large (*n* = 200). When the dimension of taxa-set increases, QCAT can quickly lose power and become less powerful than both MMD and MiRKAT (Figure 2).

Among the three more powerful adaptive tests, MAX seems to be slightly more powerful than AMDA and OMiAT when the density of signal is sparse (p^*^/*p* = 10%) and dimension is relatively low (*p* = 50,100, and 200) as indicated in Figure 1 and the top row of Figure 2. Compared to AMDA, MAX only utilizes the strongest signal, which could be beneficial when the signals are extremely sparse. When *p* = 500, there are p^*^ = 50 even under the sparse scenario and AMDA can be more powerful than MAX by including more signals in the testing subset (bottom row of Figure 2). On the other hand, when the signal level is moderate (p^*^/*p* = 30%) or relatively dense (p^*^/*p* = 50%), AMDA is much more powerful than MAX under most scenarios in both Figures 1, 2. Finally, as seen from both figures, AMDA is always more powerful than OMiAT across all scenarios. AMDA and OMiAT treat variables in different ways. AMDA selects some variables and excludes the rest for further subset testing, while OMiAT assigns different weights for different variables when calculating the multivariate score test statistic. Despite that a small non-zero weight may be assigned to a noise variable in OMiAT, due to the relatively sparse signal density (p^*^/*p* ≤ 50%, which means there are much more noises than signals), the accumulated adverse effects of noise variables can still deteriorate the performance of OMiAT. As a comparison, a zero weight is assigned to a noise variable (by excluding it from the testing subset) in AMDA, which explains power gain in AMDA over OiMAT.

To conclude, like five other methods, the proposed AMDA method is able to preserve the nominal type I error in microbiome differential abundance analysis. Power-wise speaking, there is no uniformly most powerful test in our simulations. However, the proposed AMDA method is always the most powerful one among all six tests being evaluated in this simulation under most scenarios, and the power advantage of AMDA over the other five methods can be huge (Figures 1, 2). Under only a few particular scenarios with extremely sparse signal (p^*^/*p* = 10%) under relative low dimensions (*p* = 50,100, and 200), MAX can be slightly more powerful than AMDA.

### 3.3. Application to Oral Microbiome Data Collected From Children With Autism Spectrum Disorder

We applied the proposed AMDA method to a study investigating how the oral microbiome differs across children with autistic behaviors (Hicks et al., 2018). The study enrolled 346 children (between 2 and 6 years old), which were divided into three groups according to the severity of disorder/developmental status: autism spectrum disorder (ASD, *n* = 180), non-autistic developmental delay (DD, *n* = 60), and typically developing (TD, *n* = 106). The ASD group was defined using criteria specified in the Diagnostic and Statistical Manual of Mental Disorders (DSM–5) by the American Psychiatric Association. The DD group included children who did not meet DSM-5 criteria for ASD but had developmental delay symptoms (e.g., expressive speech delay and intellectual disability). TD children included children with negative ASD screening and met typical developmental milestones on standardized physician assessment. The oral microbiome composition of these children was quantified with next generation sequencing. The data along with details of data processing are available in the previous publication (Hicks et al., 2018).

Taxonomic reads were further filtered to include only the taxa with counts of more than 10, in more than 20% samples, which ended up with a oral microbiome community of 753 taxa. Sequence alignment with the k-SLAM (Ainsworth et al., 2017) method was used for comprehensive taxonomic classification, and these 753 taxa were classified into 457 species, 266 genera, 142 families, 73 orders, 33 classes, and 16 phyla (each rank had a Unclassified group for taxonomic sequence not identified at that rank). Because the proposed AMDA method is an adaptive multivariate two-sample test, we focused our analysis on higher taxonomic ranks (family, order, class, phylum, and the community of all 753 taxa), as many lower taxonomic ranks contain only a single taxon (e.g., 410 of the 457 species are a singleton). Similarly, for the taxonomic ranks (family, order, class, and phylum) being considered, we further limited our analysis to a particular taxa-set that contains more than two taxa. As a result, 52 families, 34 orders, 18 classes, and 10 phyla were tested in our data analysis. We applied AMDA, MAX, OMiAT, MMD, MiRKAT, and QCAT to this data to examine the oral microbiome differences among three different children developmental profile groups (particularly, ASD vs. DD and ASD vs. TD) at different taxonomic ranks. As 52 families/34 orders/18 classes/10 phyla were tested, we adjusted for multiple testing using the Bonferroni correction to control the family-wise error rate at α = 0.05. Correspondingly, *B* = 10, 000 permutations/resamplings were used in AMDA, MAX, OMiAT, MMD, and QCAT to increase the precision of the test *p*-values, while the MiRKAT calculates the *p*-value analytically.

We first applied these tests to examine whether there is an overall shift in oral microbiome composition between different developmental groups by testing the differential abundances of all 753 taxa as a whole community. For the comparison of ASD vs. DD, the test *p*-values of AMDA, MAX, OMiAT, MMD, MiRKAT, and QCAT are 0.0113, 0.1409, 0.5244, 0.1321, 0.1377, and 0.9802, respectively. AMDA is the only method that is able to detect a significant (*p*-value < 0.05) difference of microbiome community profiles between ASD and DD. For the comparison of ASD vs. TD, the test *p*-values of AMDA, MAX, OMiAT, MMD, MiRKAT, and QCAT are 0.0021, 0.0017, 0.0323, 0.3039, 0.3099, and 0.1782, respectively. All three adaptive methods (AMDA, MAX, and OMiAT) are able to detect a significant difference between ASD and TD. In the original study (Hicks et al., 2018), the Mann-Whitney *U*-test based individual differential analysis was applied to each taxon and only three/six taxa were differentially abundant between ASD vs. DD/ASD vs. TD under FDR = 0.05 [see Table 2 of Hicks et al. (2018)]. According to the previous simulation results, when the number of signals is relatively small (p^*^ = 3 or 6 as suggested in the original analysis) compared to the number of variables (*p* = 753), the non-adaptive tests have a low power. This explains that MMD/MiRKAT/QCAT methods are not able to detect a significant difference of microbiome profiles between two conditions in this data. Finally, the AMDA/MAX/OMiAT *p*-value of comparison ASD vs. TD is much smaller than that of comparison ASD vs. DD, indicating a more significant overall oral microbiome composition difference between ASD vs. TD than the between ASD vs. DD, which is consistent with the severity of disorder.

**Table 2:** Number of significant differential abundant taxa-set at each taxonomic rank detected by different methods under family-wise error rate of 0.05.

**Comparison**	**Rank**	**AMDA**	**MAX**	**OMiAT**	**MMD**	**MiRKAT**	**QCAT**
	Phylum (10)	3	1	0	0	0	1
ASD vs. DD	Class (18)	3	1	1	2	2	1
	Order (34)	2	0	0	1	1	2
	Family (52)	1	0	0	0	0	1
	Phylum (10)	2	2	2	0	0	1
ASD vs. TD	Class (18)	4	3	3	2	2	5
	Order (34)	3	2	1	1	1	2
	Family (52)	2	2	1	2	2	2

*Number in parentheses denotes the total number of tests conducted at that rank*.

**Figure 3:**
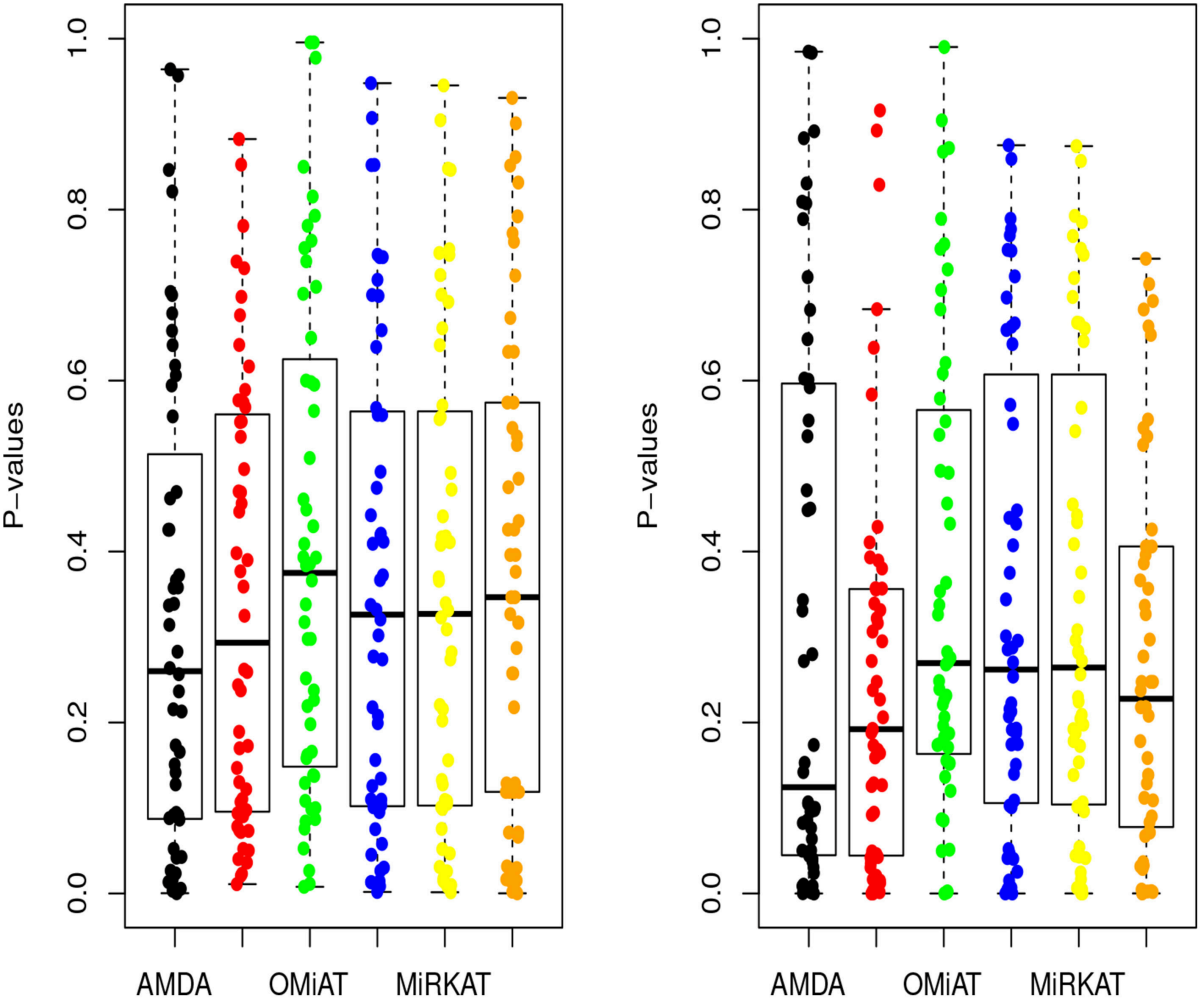
*P*-values of AMDA, MAX, OMiAT, MMD, MiRKAT, and QCAT for family-level differential abundance analysis. The left panel corresponds to the comparison between ASD and DD, and right panel corresponds to the comparison between ASD and TD.

Next, we shift our analysis unit to lower ranks than the community-level to comprehensively assess taxa-set (with multiple taxa) at each taxonomic rank that are differentially abundant among different developmental status groups. The testing results are summarized here in Table 2. Based on this table, one can observe that the proposed AMDA always declares more significant differences than the other two tests except for one scenario (class-level differential analysis between ASD and TD). The absolute difference among three methods presented in Table 2 may be small due to the conservativeness of the Bonferroni correction. To observe the relative trends of different tests, the *p*-values of these tests at family-level are presented in Figure 3 (*p*-values at other taxonomic ranks have the similar pattern and hence are not reported). The AMDA *p*-values tend to be the smallest among *p*-values of all six tests. Therefore, our method has a clear advantage over the other methods in terms of detecting more significant differences in this oral microbiome data differential abundance analysis.

## 4. Discussion

With the ever-increasing availability of microbiome and metagenomics data generated by next generation sequencing technology, the need to develop and implement efficient statistical analysis for the data is important to ensure both statistical rigor and biological relevance. In this paper, we consider the problem of differential abundance analysis for microbiome data, which leads to a better understanding of the behavior of microbiome communities. Most existing methods tackle this problem using individual taxon-based approach followed by multiple testing adjustment. However, as taxa living in the same community do not grow independently, the complicated interactions among taxa result in complicated correlation structures among taxa relative abundances, which may violate the correlation assumptions (among individual tests) of existing multiple correction methods (Hawinkel et al., 2017). On the other hand, the newly proposed AMDA examines the differential abundance of a taxa-set typically containing taxa from the same genus/family/order/class/phylum, which provides an invaluable compliment to the individual taxon-based differential abundance analysis. Given evidence of an association of a taxa-set with the outcome and assuming that at least one outcome-associated taxon within the set exist, applying AMDA to a high taxonomic rank can provide a useful preliminary screening of the whole microbiome (all species in the community) and facilitate more targeted downstream laboratory-based microbiome fine-mapping and functional studies (Wang and Jia, 2016).

The AMDA method has two main advantages compared to a traditional individual taxon-based approach. First, it can provide new biological and biomedical insights. The joint modeling of all taxa in the set is able to capture conditional effects of taxa that are missed in the traditional individual taxon-based approach, and thus new insights can be gained by shifting the analysis unit to a higher taxonomic rank. Second, it is statistically powerful by aggregating marginal signals of individual taxon and reducing the multiple testing burden. By adaptively choosing the subset being tested, our AMDA further boosts the statistical testing power compared to existing taxa set-based differential abundance analyses (e.g., MiRKAT). Moreover, the adaptive strategy used in AMDA could be easily extended to other hypothesis testing framework (e.g., association testing) beyond the two-sample problem considered in this paper. We conducted comprehensive numerical simulation studies to show the superior performance of AMDA over existing approaches in terms of maintaining the correct type I error while having a higher power to detect a true difference. The potential usefulness of AMDA was further demonstrated via its application to an oral microbiome data, where AMDA tends to detect more significant differences than its competitors.

For illustration of our method, we applied the Gaussian kernel-based MMD test, which has been shown to be a consistent two-sample test (Gretton et al., 2007, 2012). The numerical performance of AMDA using other kernels including Unifrac and Bray-Curtis (Zhao et al., 2015) is similar to the one based on the Gaussian kernel (data not shown). As the field matures, more complex (such as family-based and longitudinal) study designs have become increasingly popular in the scientific community to study the association between microbiome and various clinical and biological covariates. This is partially because these advanced designs can be more efficient to control potential confounders compared to the population-based studies with unrelated individuals. The current adaptive multivariate microbiome differential abundance analysis is developed for independent samples. It is of further interest to extend it to accommodate correlated microbiome samples collected from a study using such a complex design. The current permutation-based testing subset selection procedure has been shown to have better numerical performance in terms of selecting more signals into testing subset than existing methods across a wide range of scenarios. Yet, any theoretical guarantees of this permutation-based selection procedure is largely unknown. It is also of interest to further incorporate the phylogenetic tree information into AMDA to facilitate a comprehensive microbiome differential abundance analysis besides applying AMDA to one taxonnomic rank of the tree each time. We believe these issues are of importance and warrant further investigation.

## Ethics Statement

This study involves only secondary analyses, where all the utilized data sets are published in a previous study.

## Author Contributions

KB and NZ analyzed the data, drafted the paper, prepared figures and tables, AS and LX conducted the testing subset simulations, SH and FM provided and helped analyze the oral microbiome data. RW contributed substantial expertise to improve the paper and revised the paper. XZ conceived and designed the experiments, analyzed the data, wrote the paper, and software. All authors read and approved the final manuscript.

### Conflict of Interest Statement

The authors declare that this study received funding from a National Institutes of Mental Health STTR award (R41 MH111347) to Quadrant Biosciences, Inc. Quadrant Biosciences was involved with study design, and data collection for the RNA sequencing results employed in this study's secondary data analysis (autism microbiome data). SH and FM serve on the scientific and medical advisory boards of Quadrant Biosciences Inc., and SH is a paid consultant for Quadrant Biosciences Inc. The authors declare that the research was conducted in the absence of any commercial or financial relationships that could be construed as a potential conflict of interest.
