# Supplementary material for: An Adaptive Multivariate Two-Sample Test With Application to Microbiome Differential Abundance Analysis
Source: Front Genet. 2019 Apr 24;10:350. doi: 10.3389/fgene.2019.00350 (PMC6491633; doi:10.3389/fgene.2019.00350)
Supplement: Supplementary file 1 [file Data_Sheet_1.pdf]

Supplemental Material for "An adaptive multivariate two-sample test with application to microbiome differential abundance analysis"

Kalins Banerjee[1], Ni Zhao[2], Arun Srinivasan[3], Lingzhou Xue[3], Steven D. Hicks[4], Frank A. Middleton[5], Rongling Wu[1], and Xiang Zhan[1]

[1]*Department of Public Health Sciences, Pennsylvania State University, Hershey, PA 17033, USA*

[2]*Department of Biostatistics, Johns Hopkins University, Baltimore, MD 21202, USA*

[3]*Department of Statistics, Pennsylvania State University, University Park, PA 16802, USA*

[4]*Department of Pediatrics, Pennsylvania State University, Hershey, PA 17033, USA*

[5]*Department of Neuroscience, State University of New York Upstate Medical University, Syracuse, NY 13210, USA*

April 9, 2019

This supplementary material presents additional simulation studies comparing other testing subset selection procedures to the permutation-based procedure used in the main text. Besides our permutation-based procedure, both the lasso and knockoff filter-based testing subset selection procedures are evaluated. Section 1 presents results on selection performance of different testing subset methods; and Section 2 presents the test performance of corresponding AMDA test associated with each subset selection procedure. Unless specified otherwise, the setup of simulations in this supplementary material is the same as that used in the main text.

1 Testing subset selection performance

In this section, we conducted simulation studies to compare the selection performance of different testing subset procedures, including lasso, knockoff filter and the permutation-based procedure described in the main text. We only conducted simulations under the alternative model described in the main text as there was no signal for selection under the null model. A tuning parameter in the knockoff filter method is the nominal FDR level, which was set as 0.2 or 0.8 in this simulation. The liberal FDR level 0.8 was used to technically select as many signals as possible, which probably would lead to a more powerful test as indicated by the simulation results (particularly, comparison between AMDA and MAX) presented in the main text. For simplicity, in the rest of this section, we will denote these methods as L (lasso), K2 (knockoff with nominal FDR=0.2), K8 (knockoff with nominal FDR=0.8) and P (the permutation-based testing subset selection as described and used in the main text), respectively. We compared the performance of these methods by evaluating the number of true signals being selected (true positive/TP), the number of false signals being selected (false positive/FP) and the running time (TI) over 1000 simulation replications.

For ease of presentation, we only report the selection results under $p = 50$ and 200 in Table S1. Results under $p = 100$ and 500 have a similar pattern and hence are not reported. As can be seen from Table S1, method P can select the largest number of true signals under most scenarios. In order to control the false discovery rate, the knockoff method typically selects a much smaller testing subset and misses many true signals. The performance of lasso is in between P and K. Given the relatively small sample size and the non-sparsity nature of the simulation design, none of these methods achieves even a satisfying variable selection performance. However, for the ultimate goal of testing differential abundances, a method that can select more true signals for the second stage testing is preferred as indicated by the simulation results presented in the main text. For this reason, we would expect power gain of the P-based AMDA test compared to the lasso and knockoff-based tests under most scenarios. It also should be noted that method P is much faster than the others, which is very crucial for the testing part in AMDA (see Section 2 for more details).

Table S1: Performance of testing subset selection procedures under $p = 50$ (top) and $p = 200$ (bottom). Results are averaged over 1000 replicates.

		$p^*/p = 10\%$			$p^*/p = 30\%$			$p^*/p = 50\%$		
n	Method	TP	FP	TI	TP	FP	TI	TP	FP	TI
	L	0.50	2.35	0.15	1.71	2.25	0.14	3.41	2.00	0.14
	K2	0.20	0.58	0.54	0.54	0.46	0.54	1.06	0.43	0.55
50	K8	0.40	1.64	0.50	1.17	1.41	0.50	2.49	1.37	0.53
	P	2.40	18.8	0.02	7.29	14.6	0.02	12.0	10.6	0.02
	L	0.86	3.06	1.81	3.75	4.25	0.77	9.16	5.36	0.50
	K2	0.36	0.63	1.09	1.31	0.71	0.97	3.07	0.87	0.94
100	K8	0.62	1.77	1.08	2.76	2.88	0.98	6.86	4.25	0.94
	P	2.68	19.8	0.02	8.28	15.6	0.02	13.9	11.0	0.02
	L	2.06	9.48	1.58	11.5	22.1	3.53	20.7	18.7	3.14
	K2	0.93	1.51	0.83	5.72	7.79	0.80	12.9	9.84	0.77
200	K8	1.34	3.61	0.82	8.42	14.5	0.79	17.3	15.0	0.76
	P	3.22	20.8	0.02	9.67	15.9	0.02	16.0	11.5	0.02
	L	1.06	3.79	0.15	3.76	3.85	0.15	7.47	3.26	0.15
	K2	0.28	0.61	1.88	0.89	0.63	1.86	1.73	0.51	1.86
50	K8	0.72	2.54	1.84	2.62	2.70	1.83	5.68	2.45	1.82
	P	9.74	75.1	0.05	28.8	58.6	0.05	48.5	41.7	0.05
	L	2.37	5.69	0.25	10.5	8.21	0.24	20.3	7.32	0.23
	K2	0.74	0.75	2.07	2.84	1.00	2.07	5.08	0.90	2.04
100	K8	1.54	3.02	2.07	7.31	4.97	2.09	14.9	5.17	2.05
	P	11.1	79.0	0.06	33.5	62.1	0.06	56.0	44.2	0.06
	L	6.11	12.6	0.68	26.5	22.0	0.54	44.6	18.1	0.48
	K2	2.26	1.34	2.49	9.46	2.42	2.46	16.6	2.64	2.49
200	K8	4.27	5.83	2.43	20.0	12.3	2.41	37.9	15.4	2.42
	P	12.9	82.5	0.07	38.7	64.6	0.07	64.2	45.8	0.07

2 The corresponding AMDA test performance

The running time of testing subset selection is a big concern in AMDA because the selection procedure is applied to each permutation (see Algorithm 1 in the main text for details). Taking knockoff under $p = 200$ as an example, the running time of K2/K8 is about 2 seconds (Table S1), and as a result, it takes about 2800 computational hours to implement K2/K8 to 5000 replicates with each having 1000 permutations. As a comparison, it only takes about 70 hours to evaluate P-based AMDA to the same datasets. Given the huge computational burden of K2 and K8, we use $B = 200$ (rather than 1000) permutation to calculate the AMDA test p-value in this section. The new computing time is about 1200 hours in total (for L, K2, K8 and P), which is tolerable if we run the computer simulations in parallel. The type I error rates of different testing subset selection procedure-based AMDA tests are reported in Table S2. As can be seen from the table, all tests have the correct type I error rate.

Table S2: Empirical type I error rates of AMDA based on different testing subset selection procedures. Results are averaged over 5000 replicates.

p	n	L	K2	K8	P
	50	0.0468	0.0494	0.0354	0.0512
50	100	0.0498	0.0492	0.0420	0.0520
	200	0.0474	0.0502	0.0406	0.0512
	50	0.0522	0.0530	0.0406	0.0452
200	100	0.0508	0.0486	0.0426	0.0462
	200	0.0556	0.0536	0.0432	0.0542

The power of the tests under $p = 50$ and $p = 200$ are reported in Figure S1. The AMDA test based on the permutation-based testing subsets is consistently more powerful than the counterparts based on lasso and knockoff. The power difference between the proposed P-based AMDA test and a lasso/L-based or knockoff/K-based AMDA test is substantial when the signal is relatively dense (i.e., $p^*/p = 30\%$ or 50%). which is similar to the comparison between AMDA and MAX observed in the main text. To conclude,

the proposed permutation-based testing subset selection procedure tends to select more signals than traditional variable selection methods, which leads to a much more powerful AMDA test when the signal is not very sparse.

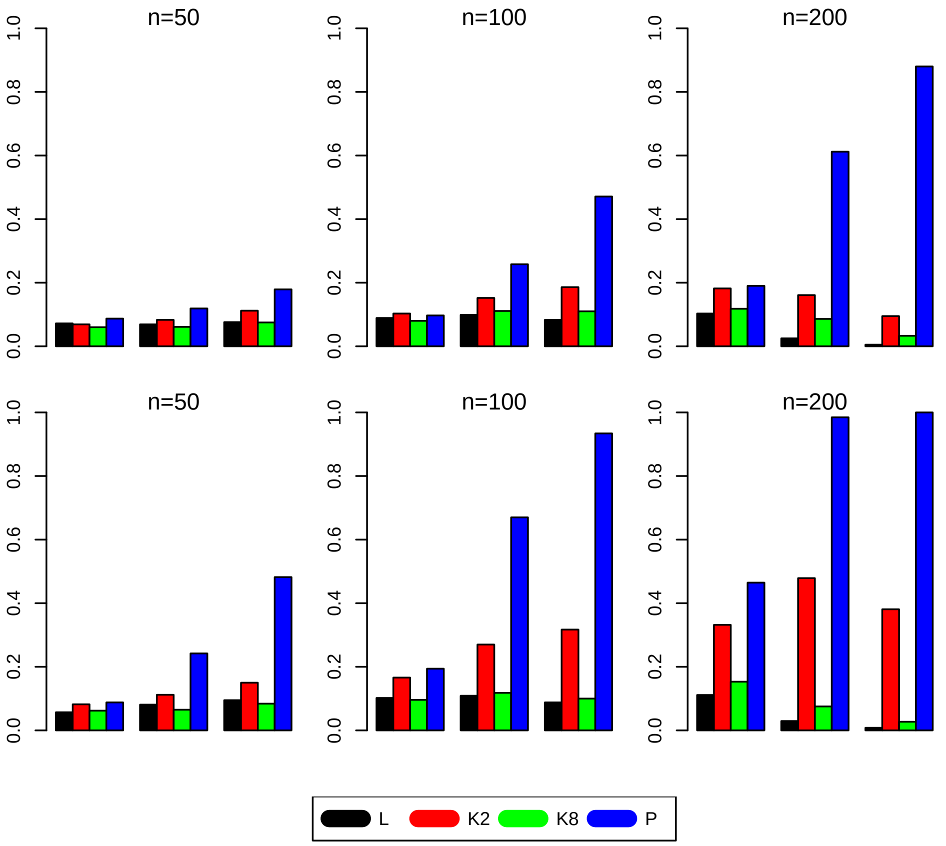

Figure S1: Empirical power of AMDA tests with different testing subsets under $p = 50$ (first row) and $p = 200$ (second row). The Y-axis represents the power and the X-axis represents the sparsity level at 10%, 30% and 50%.
